# Species Composition and Community Structure of Dung Beetles Attracted to Dung of Gaur and Elephant in the Moist Forests of South Western Ghats

**DOI:** 10.1673/031.007.5601

**Published:** 2007-11-14

**Authors:** K.V. Vinod, Thomas K. Sabu

**Affiliations:** Litter Entomology Research Unit, P.G. & Research Department of Zoology, St. Joseph's College, Devagiri, Calicut, Kerala, India 673008

**Keywords:** *Caccobius gallinus*, *Drepanocerus setosus*, *Liatongus indicus*, *Onthophagus lemniscatus*, *Onthophagus andrewesi*, *Onthophagus madoqua*, *Onthophagus devagiriensis*, *Onthophagus ensifer*, *Onthophagus castetsi*, *Onthophagus elongates*, *Onthophagus vladimiri*, *Onthophagus laevis*, *Onthophagus brutus*, *Ochicanthon laetum*, *Sisyphus neglectes*, *Elephas maximus*, *Bos gaurus*

## Abstract

The community structure of dung beetles attracted to dung of gaur, *Bos gaurus* (H. Smith) (Artiodactyla: Bovidae) and Asian elephant, *Elephas maximus* Linnaeus (Proboscidea: Elephantidae), is reported from the moist forests of Western Ghats, in South India. The dominance of dwellers over rollers, presence of many endemic species, predominance of regional species and higher incidence of the old world roller, *Ochicanthon laetum*, make the dung beetle community in the moist forests of the region unusual. The dominance of dwellers and the lower presence of rollers make the functional guild structure of the dung beetle community of the region different from assemblages in the moist forests of south East Asia and Neotropics, and more similar to the community found in Ivory Coast forests. The ability of taxonomic diversity indices to relate variation in dung physical quality with phylogenetic structure of dung beetle assemblage is highlighted. Comparatively higher taxonomic diversity and evenness of dung beetle assemblage attracted to elephant dung rather than to gaur dung is attributed to the heterogeneous nature of elephant dung. Further analyses of community structure of dung beetles across the moist forests of Western Ghats are needed to ascertain whether the abundance of dwellers is a regional pattern specific to the transitional Wayanad forests of south Western Ghats.

## Introduction

Dung beetles are a conspicuous component of the diversity of insects in Afrotropical rain forests (Hanski 1983; Hanski and Cambefort 1991; Hanski and Krikken 1991; Davis 2000a; Escobar 2000; Feer 2000; Estrada and Coates-Estrada 2002; Scheffler 2005). They use dung produced by forest vertebrates, particularly mammals and occasionally that of birds and reptiles (Howden and Young 1981; Hanski and Cambefort 1991; Estrada and Coates-Estrada 2002; Krell et al. 2003) as food and as a substrate for oviposition (Halffter and Edmonds 1982; Hanski 1989; Gill 1991). The presence of a variety of dung-producing mammals has effects on the relative abundance and diversity of dung beetles (Cambefort and Walter 1991; Hanski 1991; Estrada et al. 1999). Since, such resources can be extremely patchy in space and time, resource partitioning and competition between co-occurring species plays a major role in structuring dung beetle communities (Hanski 1991; Feer and Pincebourde 2005). Based on their nesting strategies, dung beetles are divided broadly into three functional groups *viz*., rollers (telecoprid nesters), tunnelers (paracoprid nesters) and dwellers (endocoprid nesters) (Cambefort and Hanski 1991). Rollers form food balls from a dung pat, which are rolled away, build a tunnel and bury it for use in feeding and breeding. Tunnelers create underground chambers beneath dung pat and construct nests using dung from the pat whereas dwellers breed in the dung pat itself. This functional stratification allows dung beetles to minimize the intense competition for limited food and space and also to protect the food from adverse environmental conditions (Halffter and Edmonds 1982; Cambefort and Hanski 1991).

Dung beetles have a variety of effects on the ecosystem. By burying dung and carrion as food for their offspring, dung beetles may increase the rate of soil nutrient cycling (Halffter and Mathews 1966; Bornemissa and Williams 1970; Nealis 1977) and reduce egg and larval populations of parasitic flies present in fresh dung of mammals (Bergstrom et al. 1976). Many act as important secondary dispersal agents for seeds of several tree species defecated by frugivorous vertebrates, thus participating in the natural process of forest regeneration (Estrada and Coates-Estrada 1991; Feer 1999; Vulinec 2000; Andresen 2001, 2002, 2003, 2006; Andresen and Levey 2004). In addition, they are good indicators of the impact of large herbivore and human induced change in forest habitats (Howden and Nealis 1975; Klein 1989; Favila and Halffter 1997; Davis et al. 2000; Davis 2OO0b; Davis et al. 2001; McGoch et al. 2002; van Rensburg et al. 1999; Davis et al. 2004; Botes et al. 2006).

Organization of dung beetle communities is very sensitive to changes in abundance of food resources, vegetation structure, microclimatic variables and soil characteristics (Nealis 1977; Halffter et al. 1992; Lumaret et al. 1992; Osberg et al. 1994; Davis 1996; Estrada et al. 1999; Escobar 2000; Davis 2002). Changes in community organization of dung beetles include changes in species richness, species composition, abundance and guild structure (*e.g.*, according to their diet and their resource-relocation behavior). Dung beetle communities are strongly influenced by dung type and they change in relation to the availability of different dung types (Lumaret et al. 1992; Davis 1994; Davis 2002). Though many dung beetles are generalists and do not show any dung preferences, some are strict specialists with some, or various, degrees of specialization. Some dung beetles preferably select coarse fibred dung of non-ruminants, while others prefer the more fluid and fine dung of ruminants, or the odoriferous dung of omnivores (Davis 1994; Davis 2002; Holter et al. 2002; Krell et al. 2003). Dung of howler and woolly monkeys (*Alouatta* spp.; *Lagothrix* sp.) and elephants is the preferred resource for several dung beetle species (Howden and Young 1981; Peck and Forsyth 1982; Halffter and Edmonds 1982; Cambefort and Walter 1991; Estrada and Coates-Estrada 1991; Estrada *et al.*1993; Estrada et al. 1999).

Structure of vegetation is believed to be another main factor determining the organization of dung beetle communities in tropical rainforests (Hanski and Cambefort 1991; Escobar 1997; Hill 1996; Davis and Sutton 1998; Davis et al. 2000; Halffter and Arellano 2002; Scheffler 2005). From African savannahs to Neotropical forests, dung beetles are highly habitat specific and there are distinct guilds of beetles associated with forests, edges and pasture habitats. Although some species can utilize more than a single habitat type, certain species may never be found outside their preferred habitat (Scheffler 2002).

However, in total contrast to the well documented data on the composition, community structure and habitat preference of dung beetle communities from forests of Afrotropical regions, there exist no records of the dung beetle communities of moist forests in Western Ghats despite the fact that it is a global hot spot of biodiversity (Mayers et al. 2000; Bossuyt et al. 2004). The Western Ghats is the only tropical forest ecoregion of the Indian peninsula and is well known for regional variation in vegetation, rainfall patterns, topography and high levels of endemism across its entire stretch. (Nair 1991; WWF 2001). Though mammalian diversity is lower here than in other tropical hotspots, moist forests of the region support important populations of many endemic and non-endemic mammalian species displaying different degrees of feeding habits (WWF 2001), adding opportunities for the coexistence of various dung beetle species. The Asian elephant, *Elephas maximus* Linnaeus (Proboscidea: Elephantidae) and gaur, *Bos gaurus* (H. Smith) (Artiodactyla: Bovidae), are the major mega-mammalian herbivores in the moist forests of Western Ghats (Joy 1991; Sukumar 2003; WWF 2001). The main goal of this study is to gain knowledge of the composition and guild structure of the dung beetle community attracted to gaur dung in a well protected moist forest area in Western Ghats, and to compare its community structure with the beetle assemblage attracted to dung of the Asian elephant, the other major megaherbivore in the region about which data is available from earlier studies (Sabu et al. 2006).

**Figure 1.**
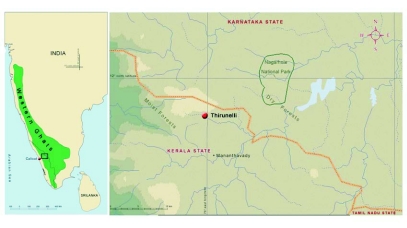
(a) Map of India showing the location of Western Ghats, (b) Western Ghats and (c) study site in Wayanad region of Western Ghats.

## Materials and Methods

### Study area

The study was carried out in Thirunelly forests (900m amsl, 20.55 km^2^) (n° 53′ N latitude and 76°01′  E longitude), 100 km North of Calicut, Kerala state (Figure 1), located in the northern boundary of south Western Ghats (WG) forests in Wayanad in the Nilgiri Biosphere region [5520.4 km^2^]. Biogeographically, Wayanad is a transition area between the moist and dry deciduous forests in south Western Ghats moist deciduous ecoregion. It harbors habitat restricted, endemic species as well as disjunct populations of species that are found in both regions (Pascal 1988; Rodgers and Panwar 1988; WWF 2001). Moist forests of the region are the summer refuge for herds of elephants and gaurs from the dry eastern side as the open grasslands, and streams originating from the upper ranges, together with the abundance of bamboo culms (*Bambusa sp.*) provide a wide choice of resource materials for grazers and browsers (Joy 1991; Nair 1991; WWF 2006). Temperature varies annually between 24–32°C. Relative humidity is in the range of 40–80%. Rainfall averages between 3,000 and 3,250 mm per year and occurs mostly in the wet months of June to November. June, July and August have the most rain (KSEB rainfall data 2002–2004). Occasional summer showers occur in April and May. Topographic variation is moderate with hills rising gently from the lower river valleys and slopes reaching 35–60°.

**Figure 2.**
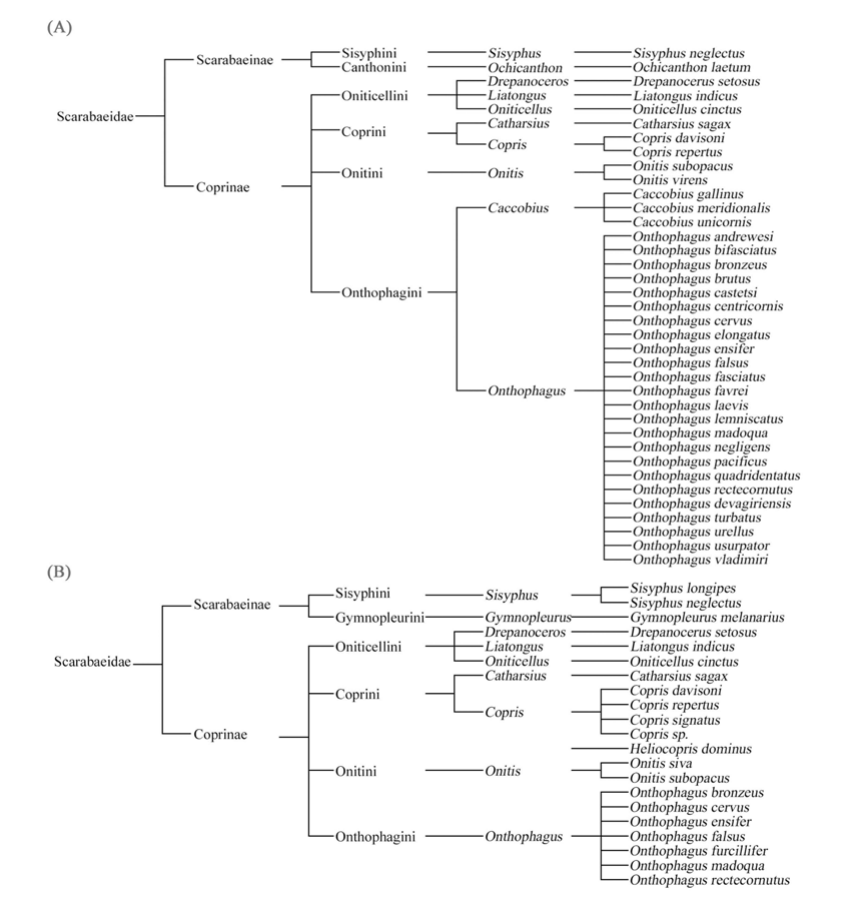
Dendrogram showing phylogenetic relationship of dung beetles attracted to gaur (A) and elephant (B) dung in the moist forests of south Western Ghats in Wayanad region.

### Methodology

The study was conducted after the rainy season between November 25, 2003 to February 2, 2004. Dung beetles were collected using Cebo-Suspendido-Rejilla pit fall traps (Lobo et al. 1988; Veiga et al. 1989; Errouissi et al. 2004). Each trap consisted of a plastic basin (210 mm in diameter and 150 mm in depth), buried to its rim in soil and containing a water-formalin-liquid soap mixture. One litre of fresh dung was placed on a strip of wire grid (2.5 cm × 2.5 cm) at the top of the basin. Each trap was topped with a dark plastic plate supported on iron bars to prevent desiccation and inundation during periods of rain. A set of four replicate traps, with each replicate at each corner of a 100 m^2^ plot was placed in the study site. The traps were collected after one week of exposure and sampling was repeated 15 times (4 traps × 15 samples). Earlier studies on the succession pattern of dung beetles showed that dung pats that were 3–7 days older attracted a subset of species that were not attracted to fresh dung (Sabu et al. 2006; personal field observation) and the gaur and elephant dung pats in the humid study region remained moist and wet for 5–7 days. Hence, sample retrieval and bait replacement was done at weekly intervals. Beetles were identified to species level using Arrow (1931) and Balthasar (1963 a, b). Identification of specimens was done by the authors and confirmed with the assistance of specialists (see acknowledgements). Beetles measuring ≥ 13 mm were considered as large (Cambefort 1991).

**Table 1.**
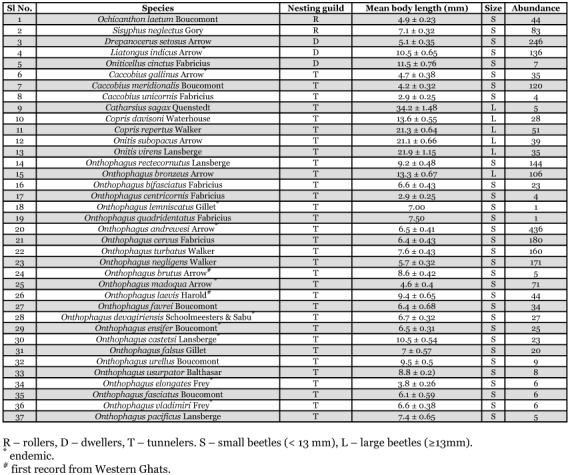
Abundance and guild structure of dung beetles attracted to gaur dung in the moist forests of Wayanad in Western Ghats.

Voucher specimens are temporarily deposited in the insect collections of St. Joseph's College, Devagiri, Calicut, and will be transferred to the national insect collections of Zoological Survey of India (ZSI), Calicut and Indian Agricultural Research Institute (IARI), New Delhi.

Rainfall data was collected from the records of Kerala State Electricity Board at Thirunelly. Humidity and forest floor temperature were assessed with thermo-hygrometer. Slope of the terrain was calculated using the trigonometric formula ‘tanθ’ (where ‘θ’ is the angle of inclination).

### Data analysis

The species diversity of the assemblage of dung beetles attracted to gaur dung pats was calculated using Fisher's alpha diversity (Fisher et al. 1943) and Simpson's dominance and evenness (Simpson 1949) indices. Beta diversity was analysed with incidence based on the Bray Curtis similarity index (Bray and Curtis 1957) as the sampling methodology employed for the collection of elephant dung beetle assemblage (Sabu et al. 2006) varied. Taxonomic diversity was analysed using non-parametric average taxonomic distinctness (Δ^+^) and variation in taxonomic distinctness (Λ^+^) indices (Clarke and Warwick 2001; Warwick et al. 2002). A regional master list of forest dung beetles from Wayanad was compiled from Sabu (2005), Sabu et al. (2006), Sabu and Vinod (2005) and the present study. A randomization test was done to detect differences in average taxonomic distinctness and variation in taxonomic distinctness, for any observed set of species, from the ‘expected’ Δ^+^ and Λ^+^ values derived from regional master species list (Clarke and Warwick 1998). Five taxonomic levels namely, species, genus, tribe, subfamily and family were considered. Branch lengths between taxonomic classes were defined following the standardization proposed by Warwick and Clarke (2001). Equal step lengths were assumed between each successive taxonomic level, setting path length ω to 100 for two species connected at the highest (taxonomically closest) possible level. So the weights used were ω = 20 (species in the same genus), ω = 40 (same tribe but different genus), ω = 60 (same subfamily but different tribe) and ω =80 (same family but different subfamily).

**Figure 3.**
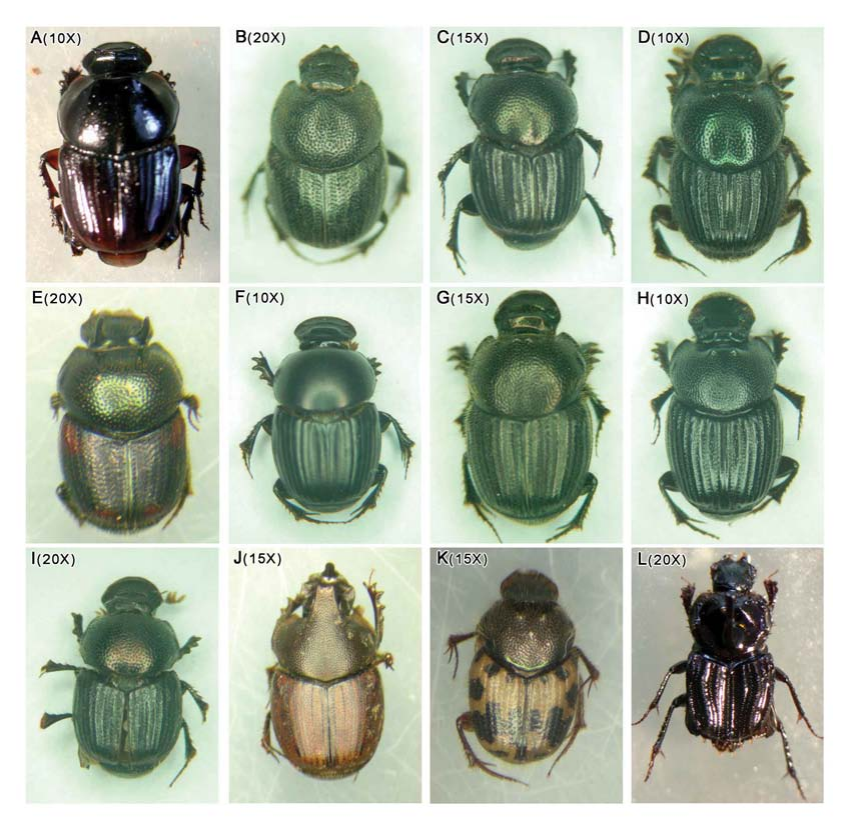
Endemic dung beetle species (A-K) and dominant dweller species (L) recorded from gaur dung baited traps from moist forests of Wayanad region of Western Ghats. (A) *Liatongus indicus* Arrow, (B) *Caccobius gallinus* Arrow, (C) *Onthophagus andrewesi* Arrow, (D) *O. brutus* Arrow, (E) *O. madoqua* Arrow, (F) *O. laevis* Harold, (G) *O. ensifer* Boucomont, (H) *O. castetsi* Lansberge, (I) *O. elongates* Frey, (J) *O. vladimiri* Frey, (K) *O. devagiriensis* Schoolmeesters & Sabu and (L) *Drepanoceros setosus* Arrow. Magnification for each image is given in parenthesis.

Simpson's diversity index was calculated with Estimates 7.5 program (Colwell 2005). All other diversity analysis was done with Primer 5 software version 5.2.9. Variances of qualitative taxonomic diversity indices values (Δ^+^ and Δ^+^) with respect to the master list values were estimated by drawing 95% confidence funnels using Primer package (Clarke and Gorley 2002). Variations in abundances among samples were analysed with one-way ANOVA test (Zar 2003). Megastat, version 10.0 (Orris 2005), was used for all statistical analysis.

## Results

As shown in Figure 2, 37 species of dung beetles representing 10 genera and six tribes were recorded. The assemblage consisted of 10 endemics (*Liatongus nidicus,                                                                                                                                      Caccobius gallinus, Onthophagus lemniscatus, O. andrewesi, O. madoqua, O. devagiriensis, O. ensifer, O. castetsi, O. elongates* and *O. vladimiri*) and two first reports from the Western Ghats (*Onthophagus laevis* and *O. brutus*) (Table 1, Figure 3). Beetles belonging to all three major functional guilds were present. *Onthophagus andrewesi*, a tunneler (18.6%), and *Drepanocerus setosus*, a dweller (10.5%), dominated the assemblage (Table 2). Tunnelers were the most speciose (32 species, 86.5 %) and abundant (78 %) functional guild. Rollers were represented by two species, *Ochicanthon laetum* and *Sisyphus neglectes*, and was the least abundant functional group (5.4 % of total abundance) (Table 1). Smaller beetles dominated the assemblage in terms of species richness (83.8%) and abundance (88.8.4%). The assemblage was moderately diverse (a = 6.63) and highly even (1-λ= 0.92). Variation in abundance among samples was not significant (df = 14, f = 1.54, *P =* 0.09). 21 species belonging to 10 genera, six tribes and three nesting guilds were collected from elephant dung (Figure 2B). Bray Curtis similarity index illustrated moderate similarity (48.28) between gaur and elephant dung beetle assemblages. Taxonomic diversity and evenness of gaur dung beetle assemblage (Δ^+^ = 42.91, Λ^+^ = 471.4) were lower in comparison to elephant dung beetle assemblage (Δ^+^ = 58.48, Λ^+^ = 344.3). Values of both taxonomic diversity indices fell within the 95% limits of the probability funnel indicating that taxonomic diversity of both the assemblages did not vary significantly from the regional species pool.

**Table 2.**
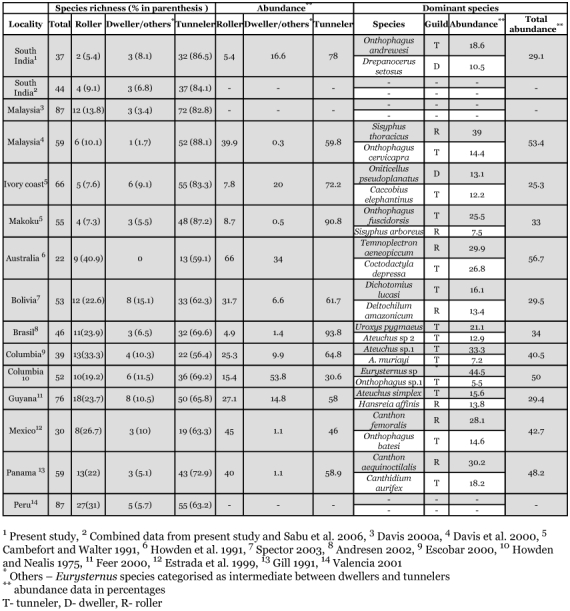
Overall species richness, guild wise abundance (%) and richness, guild structure and abundance (%) of dominant species of dung beetles in moist forests.

## Discussion

A taxonomic diversity index is a measure of biodiversity that indicates how different the species in a habitat are from each other (Harper and Hawksworth 1994). The taxonomic relatedness diversity indices have appealing sampling properties: non-dependence on quantitative data and consideration of the relatedness of species in an assemblage that are of great practical utility in diversity analysis and are considered as being most promising for biodiversity assessments (Warwick and Clarke 2001; Price 2002; Warwick et al. 2002; Magurran 2004). All dung beetle diversity assessments have been done so far with the conventional species richness and evenness-based diversity indices. However, such over reliance on patterns of dung beetle richness alone can be seriously misleading and community level data are important in dung beetle studies (Spector 2001). Hence, taxonomic relatedness based diversity properties of dung beetles attracted to dung of gaur and elephant were also used. Unfortunately, the IndVal methods of Dufrene and Legendre (1997) useful in detecting indicator species characterizing habitat types and groups of samples could not be used as the requisite data were not available.

The first report of the community structure and diversity of dung beetles in a moist forest locality in Western Ghats and South Asian region is provided. Most conspicuous is the difference observed in the guild structure of the community, when compared to dung beetle assemblages from other moist forests of the Afrotropical region. Dwellers are the dominant functional guild after tunnelers, and rollers are lower in richness and abundance. Such high abundance of dwellers is reported previously only from the moist forests of Ivory Coast in Africa.

Combining the 37 species recorded from the present study along with 7 species reported exclusively from elephant dung (Sabu et al. 2006) leads to an overall richness of 44 species in the region. Species richness is comparatively lower compared to the 87 species reported from Malaysia (Davis 2OO0a) and Peru (Valencia 2001), 76 from French Guyana (Feer 2000) and 66 species from African rain forests (Cambefort and Walter 1991). However, rain forests of Mexico (Estrada and Coates-Estrada 2002), Colombia (Escobar 2000) and Australia (Howden et al. 1991) have, on average, lower local richness of dung beetles. Though our sampling is limited to a relatively short period of two months, no additional species were added from our two year study with bimonthly random sampling from the same region with more sampling effort (Sabu 2005). Hence, we consider that present study did successfully sample most, if not all, species of dung beetles that could be trapped with baited pitfall traps from the region. Two first reports (*Onthophagus laevis* and *O. brutus*) from Western Ghats and high abundance of endemics (32.6 %) indicate that further characterization of the dung beetle faunal diversity of other forests of Western Ghats down to more local scales may reveal more details of the regional variation in endemism and localised distribution patterns.

Comparatively high abundance of old world roller, *O. laetum*, whose overall abundance is very low in south east Asian forests (Davis et al. 2000), and dominance of *D. setosus* and the endemic species *L. indicus*, are most likely a regional phenomenon. *D. setosus* and *L. indicus* are prominent dwellers in both fresh and old dung pats of elephant and gaur in the region (unpublished observations). Dominance by a few tunneler, roller or guild unspecified species (personal communications, Fernando Vaz-de-Mello) in the range of 56.7 % to 29.4% or tunneler species alone in the range of 34% 40.5%, is a general pattern of tropical moist forest dung beetle communities. Moist forests of the Ivory Coast (Cambefort and Walter 1991) and Wayanad are the only exceptions where the dominant species are distributed between tunneler and dweller guilds (29.1 % to 25.3%), and rollers are the least abundant guild.

Substantially high abundance of *D. setosus*, and L. *indicus* leads to the dominace of dwellers (Oniticellini). A similar situation exists in the moist forests of Ivory Coast in Western Africa with the abundance of *Oniticellus pseudoplanatus* (Oniticellini) and is attributed to the availability of undisturbed elephant dung pats in the region (Cambefort and Walter 1991). Dwellers are strongly associated with larger herbivore dung pats and breed successfully only in undisturbed dung pats with little competition from competitively superior tunnelers and rollers (Cambefort 1991; Hanski and Krikken 1991; Krell et al. 2003). Apparently, a similar situation prevails in the north Wayanad region with the presence of large amounts of undisturbed dung pats of elephant and gaur (unpublished observations), probably in excess of consumption by dung beetles in these forests. The moist forests of the north Wayanad region merge gently with the drier forests on the eastern slope and are a summer refuge for herds of elephants and gaurs (Joy 1991; Nair 1991). Hence we attribute the high dweller abundance in the region to the abundance and seasonal movement of large herbivorous mammals and ready availability of large dung pats.

Although dwellers are dominant over rollers in moist forests of large herbivore rich Ivory Coast and Wayanad region (Joy 1991; Nair 1991; Krell et al. 2003), we are unaware of how much the massive slaughtering of African elephants which peaked during 1980–1989 (Sukumar 2003) in Ivory Coast might have affected the availability of elephant dung and dung beetle guild structure in the region. Columbian rainforests, described earlier with high dweller abundance (Howden and Nealis 1975), showed an entirely different guild structure in more recent reports with low presence of dwellers (Escobar 2000), which is probably related to the extensive deforestation of Amazonian forests (Anderson 1990; Skole and Tucker 1993).

The low abundance of rollers is in contrast to their high abundance and richness in South East Asian forests of Borneo (Davis et al. 2000). Analysis of diversity of forest floor arthropods including dung beetles along the altitudes of Wayanad forests revealed a general low incidence of rollers and absence of large rollers above mid elevations (800m amsl) whereas both small (*Sisyphus* and *Ochicanthon*) and large rollers (*Gymnopleurus*) are abundant in the middle and low elevation (600, scorn amsl) moist forests (Sabu 2005; Sabu et al. 2006). This indicates that low presence of rollers is a regional pattern and is not a sampling error arising from the more seasonal study as in the present case. Delay in drying dung pats in shady cool forests makes dung ball making and rolling an energetically costly activity for thermophilic rollers and makes them competitively inferior to other guilds (Krell et al. 2003). Hence, the low forest floor temperature and high humidity in these shady high humid forests which keeps elephant (5–7 days) and gaur dung pats (10–15 days) moist and wet for a longer period, as was found during succession studies with elephant dung (Sabu et al. 2006) and as observed in field conditions (unpublished observations), are likely to be the major reason for the lower abundance and richness of rollers in the region.

Although, variations in sampling effort restricts our ability to interpret the data, comparison of the assemblages suggests that gaur dung attracts a more highly speciose dung beetle assemblage than elephant dung. The dominance of small sized dung beetles in both elephant (Sabu et al. 2006) and gaur dung baited traps and dung pats (unpublished observations) indicate that availability of large voluminous dung pats do not lead to an abundance of large dung beetles in the study region. Dung of the non-ruminating elephant is more fibrous and coarse than gaur dung, but they are similar in the sense that they are both herbivore in origin, moist and non-pelleted (Botes et al. 2006; Doube 1991). Moderate similarity values indicate that beetle assemblages attracted to either elephant or gaur dung do not constitute entirely dissimilar communities, but rather one community with more generalists that can use both dung types and a few specialists as well. The presence of 7 species exclusively in elephant dung baited traps, along with the categorization of dung beetles into coarse and fine dung feeders (Davis 2002; Holter et al. 2002), suggest that they are elephant dung specialists. Absence of 23 dung beetle species attracted to gaur dung in elephant dung baited traps may be related to the fluid dung preference of these species. However, variations in the sampling methodology necessitate more empirical studies to reach conclusions.

Although species richness was higher in the dung beetle assemblages attracted to gaur dung pats, high Λ^+^ (low taxonomic eveness) values indicate the presence of a phylogenetically closely related dung beetle assemblage. Analysis of taxonomic evenness by truncating the tree at various places and by removing the speciose genera showed that both taxonomic evenness and diversity of gaur dung beetle assemblage equaled that of elephant when species distribution under the genera *Onthophagus* and *Caccobius* were made even in both assemblages. High unevenness in taxonomic structure of the gaur dung beetle assemblage arises from the overrepresetation of *Onthophagus* and *Caccobius* species. The presence of 24 species of *Onthophagus* and 3 species of *Caccobius* in gaur dung (65% of the species attracted to gaur dung from genus *Onthophagus* and 73% from genus *Onthophagus* and *Caccobius)* compared to the presence of 7 *Onthophagus* species (33.3%) and the absence of *Caccobius* in elephant dung, reduced the taxonomic evenness of gaur dung beetle assemblage. This variation is distinctly shown by Λ^+^, as the variation in taxonomic distinctness index is sensitive to variations in taxonomic evenness of the assemblage and the presence of speciose genera reduces the taxonomic evenness of the assemblage which is reflected as higher Λ^+^ values.

**Figure 4.**
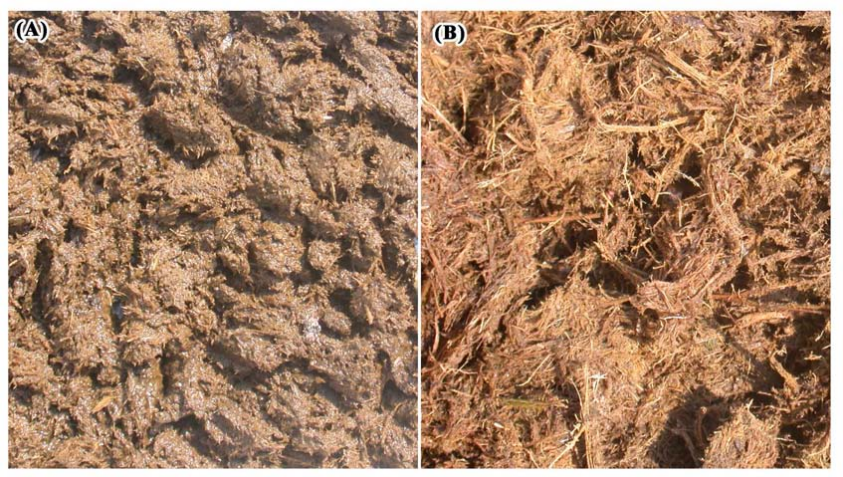
Physical structure of gaur and elephant dung from moist forests of south Western Ghats in Wayanad region, a). Fine fluid dung of gaur and b). Coarse fibrous dung of elephant.

he overrepresentation of closely related species, and the resulting high uneveness of the taxonomic struture of dung beetles attracted to gaur dung in comparison to elepahnt dung, we relate to the coarse and fine dung preferences of dung beetles (Davis 2002; Holter et al. 2002), and to variation in the physical properites of the two dung types (Figure 4). For all groups of organisms, specific taxa attain their highest diversity in particular habitats, and when certain habitat types are absent from an area some groups of species become underrepresented while others become overrepresented compared to the regional picture (Warwick and Clarke 2001), resulting in a more uneven distribution across the phylogenetic tree. Dung pats are patchily distributed and ephermeral minor habitats for dung beetles (Elton 1949; Hanski 1991). Though many dung beetles are generalists and do not show any dung preferences, some preferably select coarse fibred dung of non-ruminants while others prefer the more fluid and fine dung of ruminants, and some others the odoriferous dung of omnivores (Davis 1994; 2002; Holter et al. 2002; Krell et al. 2003). Hence, two structurally different and contrasting dung types (i.e. two minor habitats), the homogenous, fine, fluidy dung of the ruminant gaur and the hetreogenous dung of the elephant with both fibrous and fine dung particles, are readily available for the dung beetle community in the study region. Homogenous, fine gaur dung pats attract species with similar (fine) dung resource requirements and hence more closely related species belonging to specific genera or tribes. Whereas, heterogeneous elephant dung attracts both coarse and fine dung feeders and generalists from different tribes and genera (i.e. less related species) leading to the higher taxonomic evenness that is distinct in the dendrogram. The average taxonomic distinctness Δ^+^ of the assemblages showed lesser variations than Λ^+^, as Δ^+^ considers only the relatedness between individual member species involved and not the taxonomic evenness properties of the assemblage.

In summary, the present study provides for the first time data about community structure of dung beetles from moist forests of Western Ghats, as well from a South Asian region. Though with low species richness, elephant dung attracts a more taxonomically diverse and even dung beetle assemblage than gaur dung that is likely to be related to the more heterogenous physical nature of elephant dung with both fluid and fibrous dung particles. The presence of many endemics (27 %), predominance of *O. andrewesi*, an endemic of the Western Ghats, and *D. setosus* recorded only from the Indian continent, and the higher incidence of the old world roller *O. laetum*, makes dung beetle assemblage in the moist forests of this region unusual. The dominance of dwellers (Oniticellini) over rollers makes the functional guild structure of dung beetle assemblage of the Wayanad forests more similar to the dung beetle community of the Ivory Coast forests of Western Africa and different from those of south East Asian (Borneo) and Neotropical forests. Furthermore, the current study reiterates that the abundance of dwellers is an indicator of the availability of undisturbed dung pats and herbivore abundance in moist forests. However, not enough data exists to establish that the predominance of dwellers, and the low abundance and species richness of rollers, is a general pattern applicable to entire moist forests of Western Ghats. Further studies are necessary to ascertain whether it is a regional pattern specific to the transitional Wayanad forests of Western Ghats alone.
